# A Highly Compact Zip Chain Arm with Origami-Inspired Folding Chain Structures

**DOI:** 10.3390/biomimetics8020176

**Published:** 2023-04-24

**Authors:** Dong-Ki Kim, Gwang-Pil Jung

**Affiliations:** Department of Mechanical and Automotive Engineering, SeoulTech, Seoul 01811, Republic of Korea; stardust1067@seoultech.ac.kr

**Keywords:** deployable robot arm, origami-inspired robot arm, zip chain, variable stiffness, bending stiffness

## Abstract

A deployable robotic arm can be a useful tool for mobile systems to widen accessible areas without removing mobility. For practical use, the deployable robotic arm needs to satisfy two requirements: a high extension–compression ratio and robust structural stiffness against the environment. To this end, this paper suggests, for the first time, an origami-inspired zipper chain to achieve a highly compact, one-degree-of-freedom zipper chain arm. The key component is the foldable chain, which innovatively increases the space-saving capability in the stowed state. The foldable chain is fully flattened in the stowed state, allowing for storage of many more chains in the same space. Moreover, a transmission system was designed to transform a 2D flat pattern into a 3D chain shape in order to control the length of the origami zipper. Additionally, an empirical parametric study was performed to choose design parameters to maximize the bending stiffness. For the feasibility test, a prototype was built and performance tests were executed in relation to extension length, speed, and structural robustness.

## 1. Introduction

Deployable robotic arms have been actively studied to widen reachable distances while maintaining a compact form factor when the arm is not in use. Due to their capabilities of space saving and reachability, the deployable robotic arms have a variety of applicable areas. For mobile robots such as unmanned aerial vehicles (UAVs) and unmanned ground vehicles (UGVs), the deployable robotic arms improve their ability to access hard-to-reach areas and confined spaces [1,2,3,4,5,6,7,8,9].

To make a deployable robotic arm for practical use, two basic conditions are required: space-saving capability and structural stiffness against environments. To fulfill such conditions, researchers have proposed attractive ideas from the perspective of folding-based mechanisms or zipper-based mechanisms.

For the folding-based mechanisms, Kim et al. suggested an origami-inspired robotic arm that can be folded flat. The proposed arm consists of seven foldable modules connected in series and has a thickness of 40 mm in a fully folded state and 700 mm in an extended state [10]. The robotic arm has a locking mechanism using the perpendicular folding method. This locking mechanism substantially increases the bending stiffness and resistance against compression. Therefore, the robotic arm was installed on a UAV and successfully grasped an object upon approaching a confined space.

Suthar et al. employed a foldable scissor linkage as a deployable robotic arm [11]. The robotic arm can be extended from 0.07 m to 0.4 m, weighs 0.23 kg, and generates a pulling force of 0.3 kg. To make the lightweight but powerful transmission system, a twisted string actuator (TSA) was applied. In their results, the authors showed practical use of the arm by equipping it on a UAV and pulling a payload.

The other concept of the deployable robotic arm is based on the zipper-based mechanism. Collins et al. proposed a spherical robot arm called a spiral zipper mechanism [12,13,14]. The spiral zipper has a high extension–compression ratio, which allows it to be very compact when not in use. In addition, only a single-piece band is required, and the band transforms into a 3D tube shape through its unique transmission system. Thanks to these properties, the spiral zipper achieves long reach, high force, and low mass.

The Spiralift from Gala Systems Inc. uses two bands to make a stable and robust column. One is a horizontal band and has teeth at its circumference. The other is a vertical band and resembles celluloid with its perforated edges. The teeth and the perforated edges interlock with each other and finally form a rigid column. Depending on conditions, the Spiralift supports a load of up to a few tons.

The Zippermast from Geosystems Inc. was initially designed for UGVs. That is, the Zippermast is developed to achieve a compact and lightweight design. It has three steel bands rolled in each spool, and they are arranged 120 degrees apart. The bands have specially designed cuts at the edges, and those edges interlock through the unspooling process. This results in a rigid, strong, and straight column with a triangular cross-sectional area.

Li et al. proposed an inflatable robotic arm to inspect sensitive environments [15,16]. The arm uses artificial muscles to control the folding of the pneumatic actuators. The arm has 10 degrees of freedom and a reach of up to 10.1 m, and it weighs only 843 g. To show feasibility in the real world, a long-range demonstration was performed by avoiding obstacles and performing high-precision navigation.

**Figure 1:**
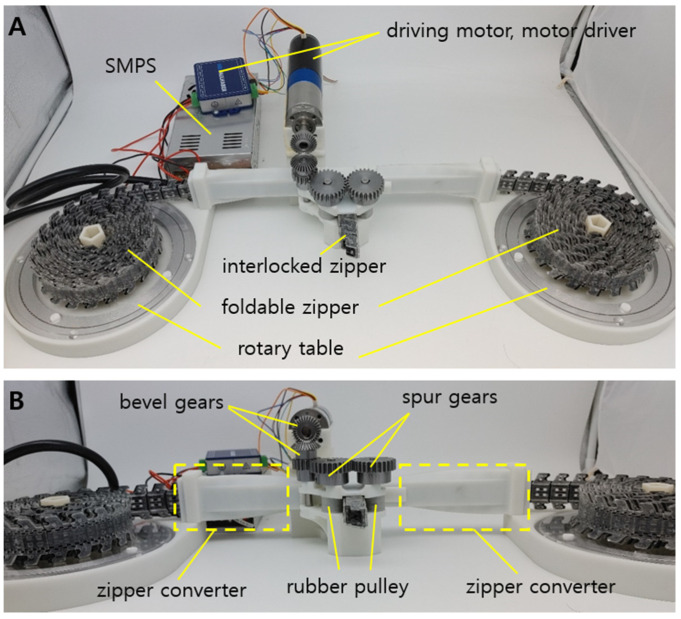
(**A**) Overall system of the origami-inspired zipper chain arm. (**B**) Transmission design for the foldable chain structures.

In this paper, we present a highly compact foldable zipper chain arm by combining the concept of both folding and zipping, as shown in Figure 1. The foldable zipper chain arm was originally inspired by the zip chain actuator (ZCA) from Tsubaki Inc. The ZCA has two chains symmetrically stowed, and they interlock with each other at the center of the actuator. The interlocked structure has high bending stiffness and resistance against compression.

The foldable zipper chain arm innovatively increases the extension–compression ratio by applying the folding process to the zipper chain. Normally, the zipper chain has a certain amount of thickness, and this thickness is maintained even when it is stowed. If the zipper chain could be flat in the stowed state, then the space-saving capacity would be substantially improved. To make this possible, the folding technique has been added to the zipper chain. Thanks to this origami-inspired zip chain and its ability to be fully flattened in the stowed position, many more chains can be stored in the same space.

In the following sections, the design, empirical parameter study, and experimental results are elucidated. The design section describes the working process of the foldable zipper chain arm. The empirical study section analyzes the bending stiffness and the maximum bending load by varying the design parameters. In the experimental results section, a prototype is built and tested to show the feasibility of the foldable zipper chain structure.

## 2. Design

### 2.1. Overall Design

The overall system consists of the foldable zipper, transmission, and electronics, as shown in Figure 1. The foldable zippers are located at each side of the system and sit on a freely rotating table. When the zippers are released, the freely rotating table reduces friction and allows the zipper to be loosened easily.

**Figure 2:**
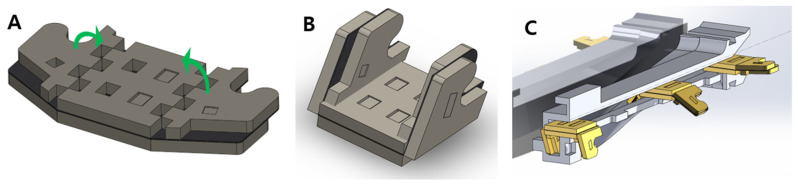
The foldable zipper. (**A**) Unfolded state and (**B**) folded state. (**C**) Zipper converter.

After the zipper is loosened from the table, the zipper passes through the zipper converter. The zippers are in the unfolded state at the beginning, as shown in Figure 2A. To be interlocked together, however, the zipper should be folded, as shown in Figure 2B. This process is achieved when the zippers pass through the converter shown in Figure 2C. In Figure 1, the zipper converter has a curved surface to smoothly convert the shape of the zipper.

The folded zipper meets its pair at the center of the system. The pairs of the zipper are joined by interlocking their teeth, as shown in Figure 4A. A detailed view of the interlocking process is shown in Figure 4B,C. The interlocked zipper is extended by the pulley-driven system. Rubber-based pulleys are employed to utilize friction between the interlocked zipper and pulleys, as shown in Figure 1B. The pulleys share an equal axis with spur gears. The spur gears are connected to the bevel gears. The bevel gear finally gets power from the motor BLDC (Faulhaber BLDC 024BS 44/1 with EN_MC5010S driver, Germany). By using the motor-powered transmission, the interlocked zipper can be extended or retracted as required.

### 2.2. Origami Zipper Design

The foldable zipper is designed to save space while it is not in use. In the case of the existing zipper chain, minimal thickness is required to incorporate both a tooth for interlocking and a joint for flexibility. To reduce the thickness, the proposed chain separates structures for interlocking and flexibility through folding.

**Figure 3:**
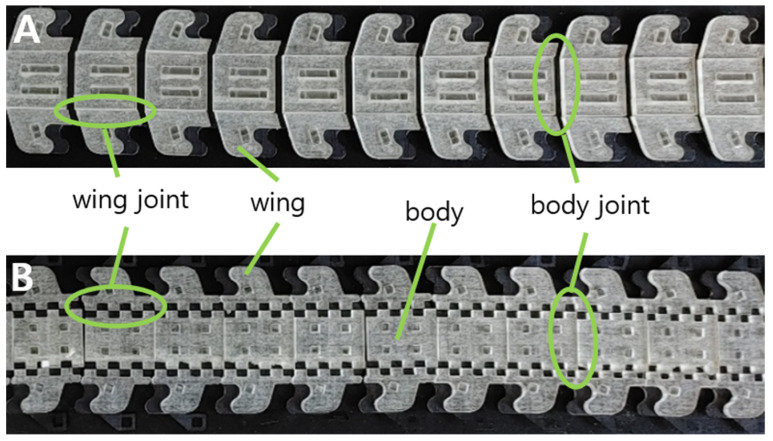
The foldable zipper. (**A**) Top side and (**B**) bottom side.

Figure 3A,B show the top and bottom of the foldable zipper. The foldable zipper consists of the body, body joint, wing, and wing joint. The role of the body joint is similar to the joint in the existing zipper chain, which gives flexibility to the chain. The main difference is the foldable wing. The wing folds and unfolds depending on the required states. The wing is in an unfolded state, as shown in Figure 2A, when the zipper is in storage. When the zipper is required to be in use, the wing is folded, as shown in Figure 2B.

**Figure 4:**
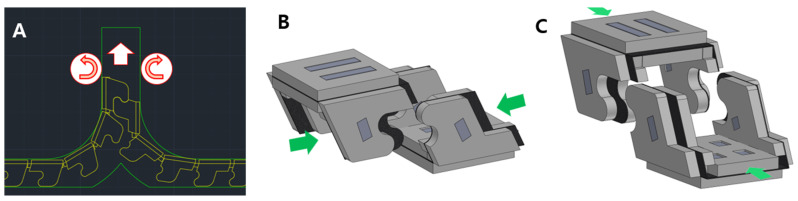
(**A**) Interlocking of foldable zipper chains. Interlocking of a single pair viewed from (**B**) the front and (**C**) the side.

The wing has a tooth-like shape and interlocks with another wing to create a stiff structure, as shown in Figure 4. The interlocking of the teeth increases the bending stiffness of the structure relative to its separated state. Furthermore, to reduce the tolerance at the wing joint and to make a stiffer structure, a castle-like pattern has been employed [17]. The castle-like pattern has an equal depth to the thickness of the foldable wing. Therefore, the wing can only be folded at exactly the right angle.

In terms of volume, the foldable wing reduces the space required to store the zipper chain. Since the zipper chain in the separated state is thin enough, the required space is substantially reduced. For example, 2100 cm^3^ of the foldable zipper chain is required to make a 5 m-long arm, while the existing chain requires 8201 cm^3^.

### 2.3. Transmission

The transmission system is based on a structure in which foldable zippers are pulled from both ends and made into a pair to launch. To pull the foldable chain from the storage table, rubber pulleys are employed. While existing zipper chain arms utilize gears, it is challenging to use gears for a foldable zipper chain because the chain is too thin to make gear teeth on the surface, which affects the strength of the foldable zipper chain.

In addition, the origami zipper is converted from a 2D shape to a 3D shape when the foldable zipper is loosened from the table for chain storage. The zipper converter is shown in Figure 1B and Figure 2C. The foldable chain enters the converter in a flat form and exits in an angled form. After this process, a pair of foldable zipper chains interlock together, as shown in Figure 4A.

In the case of storage, as the length of the foldable zipper increases, the frictional force caused by its own weight increases linearly. To solve this problem, a rotating plate is attached to the storage. The rotating plate is responsible for converting the friction between the zipper and the ground into bearing friction on the rotating plate.

When the foldable zipper is retracted and stored, the zipper must be pulled into the storing plate smoothly. To this end, a wind-up spring is installed at the central axis of the rotating plate. The wind-up spring passively retracts the foldable zipper chain, and the chain is naturally wrapped around the central axis.

### 2.4. Fabrication

**Figure 5:**
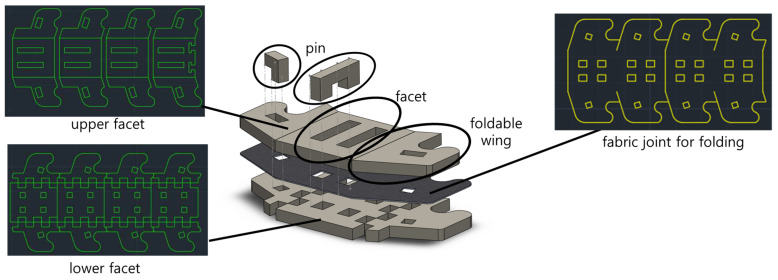
Assembly of a single module of a zipper chain to show the manufacturing process.

The foldable zipper chain is fabricated using layer-based manufacturing [18,19]. The detailed assembly process is shown in Figure 5. The foldable zipper chain has three layers: lower facet, nylon fabric, and upper facet. The lower facet and upper facet are acrylic and patterned by 2D laser machining. At the joints of the lower facets, the castle-like pattern has been applied. The castle-like pattern prevents distortion of joints from external loads. In addition, the depth of the castle-like pattern determines the range of motion of the joints. When the thickness of the lower facet and the depth of the castle-like pattern are equal, the castle-like pattern allows the joint to be folded at right angles only. In our case, they are designed to be equal to limit the folding angle of the chain. The nylon fabric acts as a joint. These layers are stacked by using double-sided tape. To prevent the stacked layers from separation, four pins pass through the whole layer, as shown in Figure 5.

**Figure 6:**
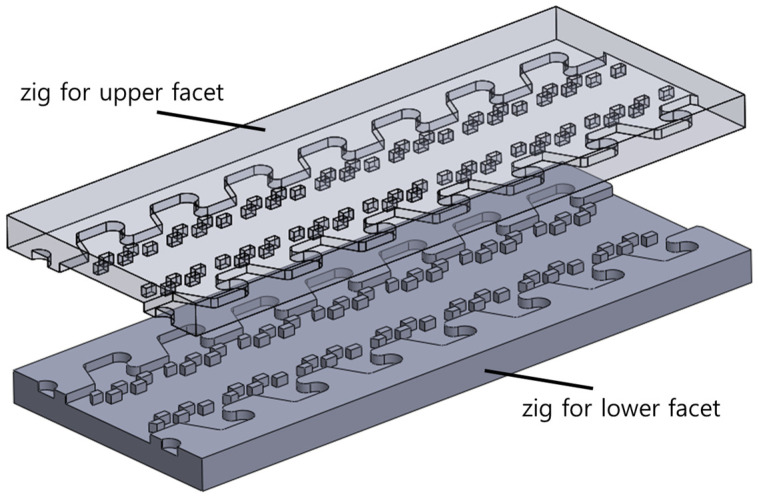
Zigs for precise assembly of the lower facet, nylon fabric, and upper facet.

Assembling a single module of the foldable zipper is easy. However, in the case of the long zipper chain, there is a high probability of tolerance, which deteriorates the performance of the foldable zipper chain. To solve this issue, specially designed zigs shown in Figure 6 have been used in the assembly process. Zigs for the upper facet and lower facet are prepared to align three layers. The lower facet and upper facet are located at their own zig and automatically aligned following the holes on the facets. In the middle of the zigs, the nylon fabric is inserted. Using this process, the foldable chain is built to be constant regardless of length.

## 3. Empirical Study on Design Parameters

### 3.1. Design Parameters

**Figure 7:**
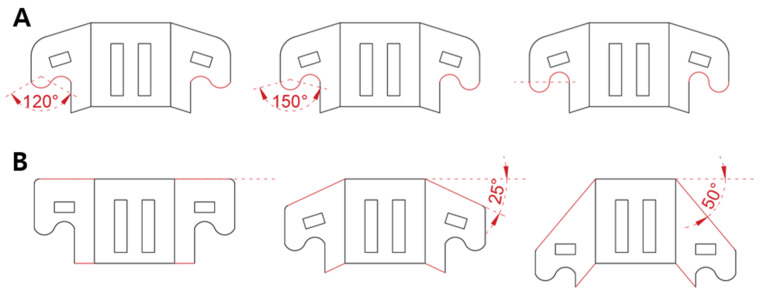
Design parameters of the foldable zipper chain. (**A**) Tooth angle. (**B**) Wing angle.

In this section, two design parameters affecting structural robustness are selected, as shown in Figure 7. The tooth angle shown in Figure 7A is related to the arc length of the tooth. As the angle increases, the shape of the tooth becomes sharper. The wing angle shown in Figure 7B indicates the inclined angle of the foldable wing.

**Figure 8:**
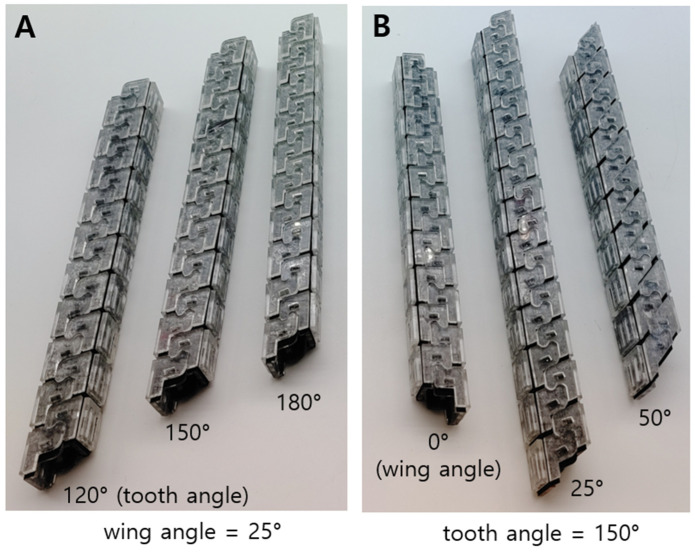
Fabricated interlocked zipper chains depending on variation of design parameters. (**A**) The tooth angle varies while the wing angle is fixed and (**B**) vice versa.

The structure shows non-linear behavior when an external force is applied. In other words, it is hard to analytically formulate the behavior. To investigate how these design parameters affect structural robustness, specimens were therefore made for the empirical study. The samples are shown in Figure 8. Figure 8A shows the samples when they have a difference in tooth angle, while Figure 8B indicates the sample when they have a different wing angle.

To check the performance of the interlocking foldable zipper chain, the bending test and compression test were performed while varying the tooth angle and wing angle. To increase the accuracy of the data, five equal specimens are prepared for each case.

### 3.2. Bending and Compression Test

The three-point bending tests and compression tests were performed to investigate the bending stiffness and compressive stiffness while varying the tooth angle and wing angle. In the case of the tooth angle, three kinds of specimens were prepared with angles of 120°, 150°, and 180°, respectively. In the case of the wing angle, three types of specimens were prepared with angles of 0°, 25°, and 50° respectively. For each case, five identical specimens were fabricated to obtain credible data.

**Figure 9:**
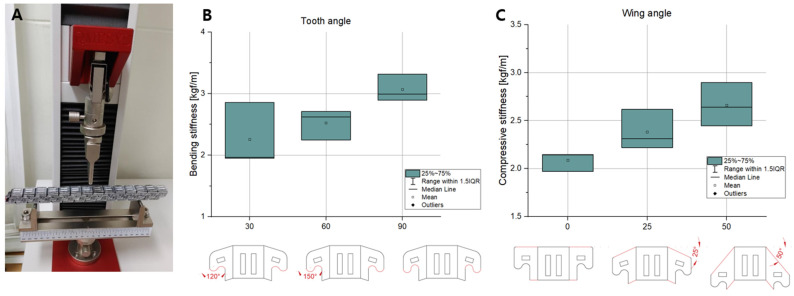
(**A**) Three-point bending test. Bending stiffness depending on (**B**) the tooth angle and (**C**) the wing angle.

**Figure 10:**
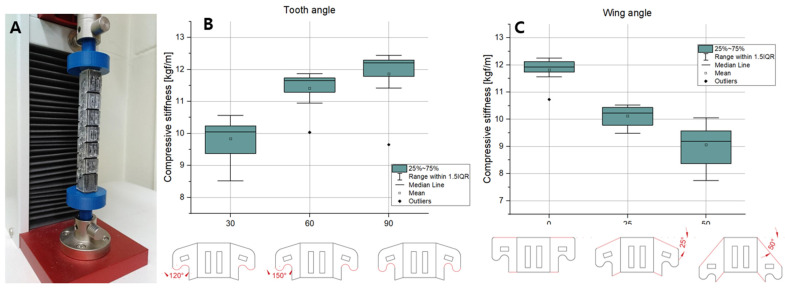
(**A**) Compression test. Compressive stiffness depending on (**B**) the tooth angle and (**C**) the wing angle.

The results are shown in Figure 9B. In Figure 9B, as the tooth angle increases, the bending stiffness tends to increase. The reason for this is thought to be that the sharper shape of the tooth angle increases the interlocking force. As the shape of the tooth becomes sharper, the area that the teeth touch increases. Consequently, the chain with a large tooth angle can withstand higher bending loads. Figure 10B shows the compressive stiffness depending on the tooth angle. As the tooth angle increases, the compressive stiffness tends to increase. The reason for this is estimated that the sharp tooth angle strengthen the interlocking force. When the tooth shape becomes sharper, the contacting area between the teeth increases. In a result, the chain with a large tooth angle can support greater compression loads.

The bending stiffness depending on the wing angle is shown in Figure 9C. As the wing angle increases, the bending stiffness seems to increase. This is presumably due to the fact that as the wing angle increases, the length of the intersecting line increases, as shown in Figure 8B. This can also be seen in Figure 7B. The length of the inclined line becomes longer as the wing angle increases, which causes an increase in the intersecting length. Through the experiment, it is shown that the chain with a large wing angle can withstand higher bending loads. The compressive stiffness while varying the wing angle is shown in Figure 10C. Unlike the bending stiffness, the compressive stiffness decreases as the wing angle becomes larger. This originated from the inclined wing shape. Figure 8B shows the interlocked chain with different wing angles. As the wing angle increases, the engaged shape of the chain changes to be closer to the vertical line. This makes it less likely to resist compression loads. For this reason, we can see that increasing the wing angle makes it vulnerable to compression loads.

## 4. Performance Tests

This section presents the results from performance tests on the origami-inspired foldable zipper chain arm. Feeding distance and speed were observed. In addition, compression load during feeding was exerted depending on the feeding distance. Lateral load was also given to check the bending during feeding.

To show the feasibility of being used in real-world applications, the foldable zipper chain arm was inserted into a pipe filled fully with grease, which is mainly used to construct nuclear power plants.

### 4.1. Feeding Distance

Feeding is the basic functionality of the foldable zipper chain arm. Through the feeding test, the successful functioning of the transmission system can be checked as well. To test the feeding of the foldable zipper chain arm, a whole system is made, as shown in Figure 1. The driving motor transmits the torque through bevel gears. The bevel gear rotates the spur gears, and the pulleys connected to the spur gears generate friction. This friction between the pulleys and the engaged chain allows the arm to extend.

**Figure 11:**
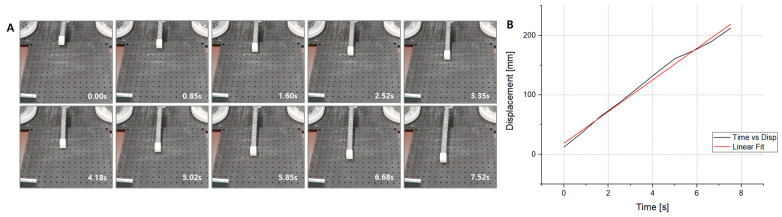
(**A**) Actuator is extending at a speed of about 4 cm/s. (**B**) Graph showing feeding distance vs. time.

The experiment was conducted until the arm extended by 300 mm. Figure 11A,B show the snapshots of the arm and the displacement, respectively. The driving test took about 8 s to achieve a 300 mm extension. The actual driving speed was given as 38 mm/s. In Figure 11B, the actual displacement has been fit with the linear formula. There seems to be a slight difference between the two lines. It is estimated that slip may occur between the foldable zipper chain and the pulleys since the system uses friction force to transmit the motor’s torque.

### 4.2. Compression and Bending Load during Feeding

**Figure 12:**
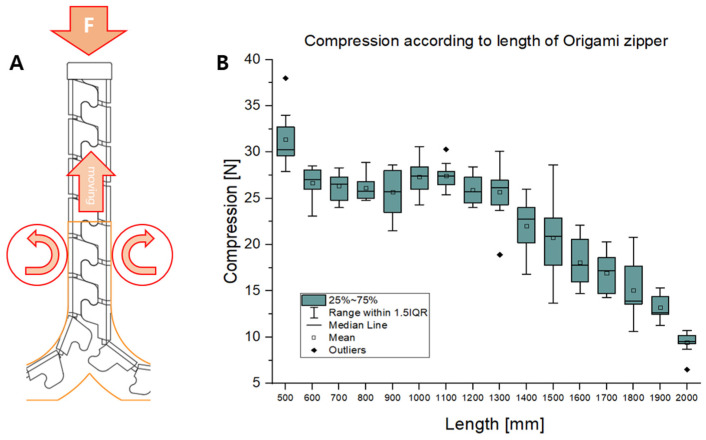
Compression load depending on the feeding distance. (**A**) Experimental setup. (**B**) Compression load while varying the extension distance.

To check the structural robustness of the foldable zipper chain arm, a compression loading test was conducted. Figure 12A shows the experimental setup. Compression load is exerted at the tip of the foldable zipper chain arm. The load is measured using a force gauge while varying the extension distance of the foldable zipper chain arm from 500 mm to 2000 mm.

Figure 12B shows the experimental results, including compression loads depending on extension distances. As shown in Figure 12B, the compression load has the highest value when the extension length is short. This is natural since the critical buckling load tends to be inversely proportional to the square of the extension length. The compression load drops to around 25 N when the extension length is 600 mm. This value is maintained until the extension distance reaches 1300 mm. When the extension length exceeds 1300 mm, the compression load starts to drop and reaches around 10 N at the extension distance of 2000 mm.

**Figure 13:**
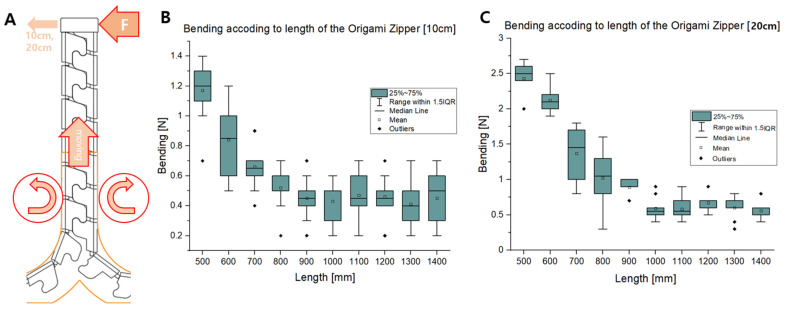
Bending load depending on the feeding distance. (**A**) Experimental setup. (**B**,**C**) Bending load while varying the extension distance.

A bending load test was also conducted to check the structural robustness of the foldable zipper chain arm. Figure 13A shows the experimental setup. The external bending load was exerted and measured while varying the extension distance of the foldable zipper chain arm. The bending load was given until the point at which the arm bends, and the leading part reaches distances of 10 cm and 20 cm.

Figure 13B,C are the experimental results, including bending loads depending on extension distances when the reaching distances are 10 cm and 20 cm, respectively. As shown in Figure 13B, the bending load has the maximum value when the extension length is short. This is natural since the bending stiffness tends to be inversely proportional to the cube of the extension length. The bending load almost linearly drops to around 0.4 N up to 900 mm. Beyond 900 mm, the load is maintained as the extension distance increases up to 1400 m. In the 20 cm case, the bending load shows a slightly higher value of around 2.5 N compared to the 10 cm case. The load drops to around 0.5 N as the extension length reaches 1000 mm. After 1000 mm, the load is maintained up to 1400 mm. In both the 10 cm and 20 cm cases, the overall trend was shown to be similar. The bending load has its maximum value at the beginning and then stiffly drops and maintains a specific value as the extension length increases.

In short, as the extension distance of the foldable zipper chain actuator increases, the compression and bending loads seem to decrease. This is because of the buckling phenomenon. Buckling easily occurs when the extension distance is long since the critical buckling load is inversely proportional to the square of the actuator length.

### 4.3. Real-World Application

The proposed foldable zipper chain arm has been developed to investigate the inside of pipes fully filled with tensioned steel wires and grease, which is used for constructing nuclear power plants. The length of the pipe typically exceeds 100 m, and an extremely long arm is therefore required to pass through the pipe. If we were to use the current mainstream model of the uncollapsible zipper chain arm, a massive amount of volume would be required to carry the whole system. The foldable chain, however, enables us to substantially reduce the required volume and to easily pack the system. In addition, one of the major advantages of the proposed chain is that there is an empty space inside the chain, and the empty space is protected from the outside environment. Therefore, critical components such as electric wires can be inserted inside the chain.

**Figure 14:**
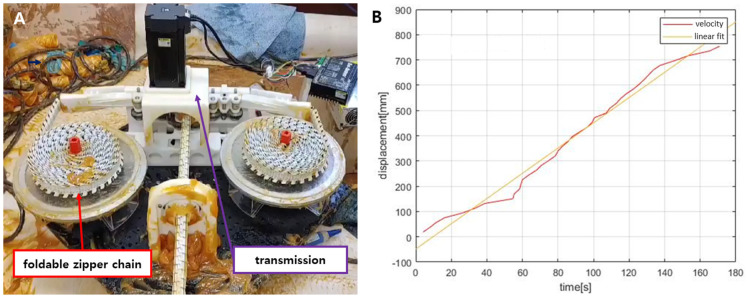
(**A**) The whole system, including an origami-inspired foldable zipper chain arm. (**B**) Extension distance vs. time.

To show the feasibility of using the pipes, the experimental setup was designed and is shown in Figure 14A. The role of the foldable zipper chain arm is to push forward the ultrasonic sensors attached to the leading part. To provide electric power to the ultrasonic sensors, several electric cables are required, which need to pass through the foldable chain arm. In this situation, the electric cables are perfectly protected from the outside environment, which is fully filled with grease. The foldable zipper chain actuator is able to extend by pushing the grease by about 0.8 m and moving at a speed of about 5 cm/s, as shown in Figure 14B. Moreover, it was shown that the grease did not penetrate the inside and that the foldable zipper had the ability to protect the internal electric cables.

**Figure 15:**
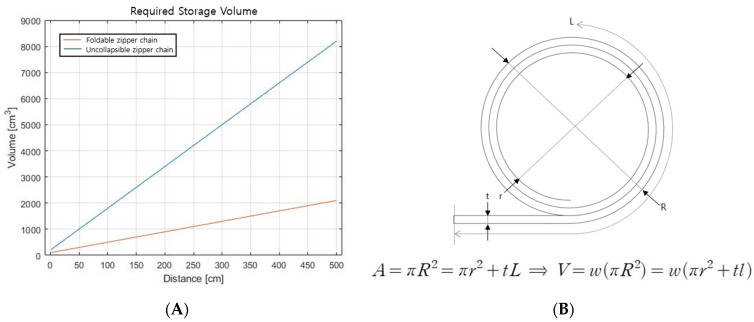
(**A**) Required storage volume for a foldable zipper chain and an uncollapsible zipper chain. (**B**) Simple model for volume calculation.

The other main advantage is the reduced required volume for chain storage. In fact, the current base frame, including transmission, seems bulky. When the length of the arm becomes much longer, however, the required volume for the arm structure can substantially decrease. Figure 15 shows the volume of storage needed depending on the length of the arm. For example, the 5 m-long foldable zipper chain arm has a volume of about 2000 cm^3^ in the stored state. If we used an existing uncollapsible zipper chain actuator, the storage volume would need to be about 8000 cm^3^, which is more than three times the volume of the foldable zipper chain arm. The storage volume is innovatively reduced, and this would prove useful in situations where workers need to carry the arm system.

## 5. Conclusions

The origami-inspired zipper chain arm, named the foldable zipper chain arm, is introduced in this article as a novel approach to achieving a highly compact arm. The foldable chain is the main element of the arm, which provides a significant advantage in terms of space saving when not in use. The foldable chain is stored in a completely flat state, enabling more chains to be accommodated in the same space. Moreover, a transmission mechanism is designed to convert the 2D flat pattern into a 3D chain shape, controlling the length of the origami zipper. This paper also includes an empirical study to determine the design parameters that optimize the bending stiffness. Finally, the feasibility of the concept was tested by building a prototype and conducting performance tests, including extension length, speed, and structural robustness tests.

There are several ways to improve the performance of the foldable zipper chain arm. First of all, the transmission needs to be modified to reduce the slippage problem during feeding. Currently, the transmission is based on the friction between the rubber pulley and the foldable zipper chain. When a large external load is exerted, there is a high probability of slippage. One of the potential solutions is to engrave the gear tooth into the foldable zipper chain arm to increase torque transmissibility. Improving structural robustness is important as well. Currently, external bending and compression loads substantially affect the behavior of the foldable zipper chain arm. To reduce this influence, other materials, such as engineering plastics, CFRP, etc., will be considered for the foldable structures.
